# The dynamic nature of crystal growth in pores

**DOI:** 10.1038/srep33086

**Published:** 2016-09-12

**Authors:** Jose R. A. Godinho, Kirill M. Gerke, Andrew G. Stack, Peter D. Lee

**Affiliations:** 1 School of Materials, The University of Manchester, M13 9PL, Manchester, UK; 2 Research Complex at Harwell, Rutherford Appleton Laboratory, OX11 0FA, Harwell, UK; 3 Chemical Sciences Division, Oak Ridge National Laboratory, PO Box 2008, MS-6110, Oak Ridge, TN 37831 USA; 4 The University of Melbourne, Department of Infrastructure Engineering, Parkville, VIC, 3010, Australia; 5 CSIRO Land and Water, Glen Osmond, PB2, SA 5064, Australia; 6 Institute of Physics of the Earth of Russian Academy of Sciences, Bolshaya Gruzinskaya 10, Moscow, 107031, Russia

## Abstract

The kinetics of crystal growth in porous media controls a variety of natural processes such as ore genesis and crystallization induced fracturing that can trigger earthquakes and weathering, as well as, sequestration of CO_2_ and toxic metals into geological formations. Progress on understanding those processes has been limited by experimental difficulties of dynamically studying the reactive surface area and permeability during pore occlusion. Here, we show that these variables cause a time-dependency of barite growth rates in microporous silica. The rate is approximately constant and similar to that observed on free surfaces if fast flow velocities predominate and if the time-dependent reactive surface area is accounted for. As the narrower flow paths clog, local flow velocities decrease, which causes the progressive slowing of growth rates. We conclude that mineral growth in a microporous media can be estimated based on free surface studies when a) the growth rate is normalized to the time-dependent surface area of the growing crystals, and b) the local flow velocities are above the limit at which growth is transport-limited. Accounting for the dynamic relation between microstructure, flow velocity and growth rate is shown to be crucial towards understanding and predicting precipitation in porous rocks.

Understanding mineral reactions in porous geological formations is currently a subject of intense study in Earth sciences as it underpins some of the most challenging environmental problems of modern society. Striking examples include the possibility of permanently sequestering CO_2_ as carbonate minerals in deep sandstone formations and in ultramafic rocks^1^,^2^,^3^, and mineral sequestration of toxic metals into the bedrock, i.e., contaminants resulting from the unconventional extraction of natural gas and oil by hydraulic fracturing^4^,^5^. Additionally, studying the effect of microstructure and the spatial distribution of precipitates within a porous rock is essential to understand the formation of ore deposits, earthquakes induced by crystallization, as well as the degradation of stone building heritage and metasomatism^6^,^7^,^8^,^9^.

Mineral reactions depend, among other factors, on the reactive surface area and ion transport at the mineral-fluid interface, which in porous media depends largely on the permeability of the porous network^10^. For example, it has been demonstrated that the dissolution rates of magnesite within a porous column depend on the local microstructure, permeability and reactive surface area^11^,^12^. These are dynamic properties that vary as the reaction modifies the pore microstructure, thus causing the reaction rates to be time-dependent^13^. For example, during mineral precipitation in a porous structure permeability decreases, which affects transport throughout the structure^13^. This time-dependency has been proposed to contribute to a key fundamental problem of modern geochemistry: the discrepancy of several orders of magnitude between reaction rates observed in the subsurface and those measured in laboratory experiments^14^.

The growth rate of a mineral phase is easier to study on free surfaces (defined here as growth without spatial restrictions) than in porous media where the range of experimental techniques that can be used *in situ* is limited. Due to the difficulties of experimentally studying the evolution of porous microstructures during growth, reactive transport modeling has been the only tool capable of providing information about the dynamics of how crystals fill the pores^15^,^16^,^17^,^18^. Still, available modelling strategies require validation against experiments that track the reaction both temporally and spatially.

Barite often precipitates during oil and gas extraction due to the mixing of sulfate rich surface water with barium rich ground water^4^,^19^. The precipitates can reduce the permeability of the reservoir, causing a decrease in extraction efficiency and increased production costs^4^,^20^. Radioactivity from radium and strontium co-precipitated with barite, is a common hazard to oil platform workers and to communities living close to hydraulic fracturing sites^21^,^22^. The ability of barite to sequester hazardous metals can reduce radium mobility in the subsurface^23^,^24^,^25^,^26^, for example, to create a reactive barrier as part of a spent nuclear fuel disposal strategy, or for removing strontium and radium from fluids resulting from hydraulic fracturing^5^. Therefore, it is imperative that the kinetics of barite growth in porous media is better studied, hence enabling an enhancement in the recovery of hydrocarbons, whilst at the same time reducing its environmental impact. X-ray computed micro-tomography (XCT) has emerged in recent years as a technique capable of quantifying mineral reactions in porous media non-destructively^27^,^28^,^29^,^30^,^31^,^32^. The high flux and high signal to noise ratio of synchrotron X-ray based imaging allow fast scans of a sample, enabling new possibilities of quantifying the changing mineral phases of a porous structure with submicron resolution as a function of time.

Using the powerful combination of 4D (3D + time) synchrotron based XCT, also named sCT, and numerical simulation of flow velocities, we study barite precipitation in a micro porous silica structure under continuous flow. The evolution of the 3D microstructure as a function of time allowed us to 1) test a dynamic model for surface area normalization of growth rates in porous media using input from sCT and an empirical equation for surface area variation as a function of time obtained for barite growth on a free surface; 2) study the growth rate variation as a function of time based on our dynamic model, which is compared with an existing static model and with growth on free surfaces; 3) relate the time-dependency of the growth rate with the changes of flow velocities, which are caused by the occlusion of pores. In summary, we study the link between the dynamic properties of a pore microstructure, such as surface area, flow velocity and permeability, and how their variation can cause important discrepancies between the growth rates measured in a porous structure and on a free surface.

## Results and Discussion

### Surface area normalization

**Figure 1:**
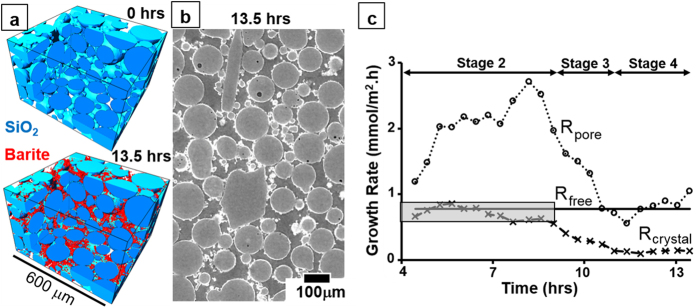
Evolution of crystals and growth rates. (**a**) 3D subvolume at the beginning and at the end of the experiment; (**b**) 2D cross section (not segmented) at the end of the experiment, where barite is the brightest fraction; (**c**) variation of the growth rate as a function of time. Dashed line (**X**), Continuous line and Dotted line (**o**) correspond to rates calculated using equation (2), measured on free surfaces for the same solution composition^33^ or calculated using the middle term of equation (1) (using *A*_*pore*_ and (Δ*V*/Δ*t*) measured here), respectively. See videos 1–2 for a full 3D reconstruction of the column.

To facilitate the understanding of the growth kinetics in porous media it is imperative to establish a method capable of linking it to the kinetics of growth on free surfaces. A major impediment to compare effectively the growth rates of a mineral phase inside a pore structure and on free surfaces is the necessity to normalize the rate to an equivalent reactive surface area. Growth rates measured on free surfaces are traditionally normalized using the surface area of the growing crystals, which can be measured experimentally^33^,^34^. However, available experimental methods do not allow the direct measurement of the surface area of crystals in pores as a function of time. Consequently, growth rates in a porous rock (*R*_*pore*_) are traditionally normalized to the total surface area of the rock, which is either measured at the beginning of the experiment^29^ or calculated geometrically^35^. However, the surface area of crystals (*A*_*crystal*_) vary with time and it’s reactivity is greater than the substrate surface (*A*_*pore*_). Therefore, for the same volume precipitated (Δ*V*) during a time interval (Δ*t*), the estimate of growth rate in porous media (*R*_*pore*_) and on free surfaces (*R*_*free*_) differ, depending on which surface area is used in the normalization, equation (1).


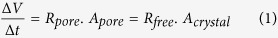


here, we use sCT to quantify *in situ* barite precipitation (Δ*V*/Δ*t*) in a microporous silica structure under continuous flow during 13.5 hours (Fig. 1). Even using sCT, the small crystal size restricts the accuracy at which *A*_*crystal*_ and its time-dependency can be measured directly^36^,^37^. Therefore, we developed a novel dynamic method of estimating the time-dependent surface area (*A*_*crystal*_(*t*)) of barite crystals using input from sCT and based on empirical equations used to calculate the surface area of barite crystals grown on free surfaces^33^. The result is a time-dependent growth rate in porous media (*R*_*crystal*_ as defined in equation (2)) that can be directly compared to *R*_*free*_. The ability to analyze the transient stages of growth and to compare *R*_*crystal*_ and *R*_*free*_ is an essential progress towards the understanding of the fundamental mechanisms affecting crystal growth in porous media, which can enable the development of dynamic kinetic models that cannot be derived from traditional experimental approaches.


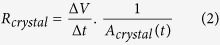


Our method of estimating *A*_*crystal (*_*t*) (units of L^2^) consists in multiplying the time-dependent surface area of single crystals (*A*(*t*), units of nm^2^/crystal) by the estimated total number of crystals in the entire pore structure, equation (3). *A*(*t*) is determined empirically, equation (4), *t* in hours. The estimated total number of crystals corresponds to the nucleation density, *N* (crystals/μm^2^), multiplied by the total pore surface area, *A*_*pore*_ (units of L^2^). To account for the specific situations where a crystal completely fills a pore space or laterally overlaps with an adjacent crystal, and thus cannot grow more in a specific direction; *A*_*pore*_(*t*) is calculated for every scan using input from sCT by subtracting the surface area of pore-throats that are filled with crystals from *A*_*pore*_ (Supplementary Fig. S1). Therefore, the area calculated using equation (3) only accounts for the surface area of crystals along the directions not restricted in space that are inherent to growth in porous media.


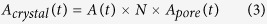



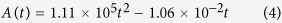


Equation (3) is based on the hypothesis that a) *R*_*free*_ = *R*_*crystal*_, thus the growth rate is not affected by the properties of the pore structure, b) on glass beads and for the solution composition and temperature of this experiment the nucleation density is constant (*N* = 0.29 crystals/μm^2^)^29^; c) the fluid composition is approximately homogeneous throughout the length of the column, thus the growth rate is the same for every crystal. The value of *R*_*free*_ = 0.78 mmol/m^2^.h was obtained experimentally during growth of barite crystals on free surfaces in a solution with the same composition used here and was normalized to the surface area of the growing crystals^29^.

### Growth rates as a function of time

Barite crystals nucleate on the surface of SiO_2_ particles growing throughout the length of the experiment (Video 1), which causes a continuous alteration of the pore microstructure (Supplementary Fig. S3). After 13.5 hours, 18% of the initial pore space is filled with barite. At the end of the experiment barite crystals can be found throughout the entire pore space (Fig. 1, Video 2) without significant variation between the center and the outer edges of the column, and between the inlet and the outlet (Supplementary Fig. S4). This suggests that at the millimeter scale, growth rates are similar throughout the length of the column. Therefore, we conclude that the ions consumed by crystal growth within the fluid residence time inside the pore network is insufficient to affect the growth rates, which is necessary to validate equation (3). Homogeneous nucleation in solution was not observed as expected^29^.

Next, we establish the link between the growth rate previously measured on free surfaces^29^ with the growth rates derived from this experiment (graphic in Fig. 1), either normalized to the initial pore surface area, *R*_*pore*_, traditional static model using the middle term in equation (1); or normalized to the surface area of the crystals quantified statistically, *A*_*crystal*_(*t*), dynamic method using equation (2). Four stages are identified. Stage 1 (initial 4 hours), barite crystals are too small to be accurately distinguished from noise. This is expected for a 1.24 μm voxel size and the growth rate of barite on free surfaces^29^. Stage 2 (4–8.5 hours), *R*_*crystal*_ (dashed line, **X**) and *R*_*free*_ (continuous line) are similar and approximately constant. In contrast, *R*_*pore*_ (dotted line, **o**) oscillates over 100% of its initial value and is significantly higher than *R*_*crystal*_. Stage 3 (8.5–11 hours), *R*_*crystal*_ progressively decreases down to five times lower than *R*_*free.*_ Stage 4 (11–13.5 hours), *R*_*crystal*_ remains approximately constant, whereby *R*_*crystal*_ = 0.14 mmol/m^2^.h.

Results from Fig. 1 suggest that the method proposed to calculate the time-dependent reactive surface area is valid until the end of Stage 2, thus validating the hypothesis that the growth kinetics on free surfaces can be applied to predict the initial stages of growth in a permeable porous structure. In contrast, using the traditional method of normalizing the growth rates to the static value of surface area of the pore structure causes a significant discrepancy between *R*_*pore*_ and *R*_*free*_ (see in Supplementary Figure S5 how this can affect the predictive calculation of crystal volumes precipitated). Similar to what has been shown for dissolution^12^, we suggest that this discrepancy can contribute to the wider geochemical problem that mineral reaction rates observed in nature do not agree with those measured in laboratory by several orders of magnitude^14^. We also suggest that more accurate methods of calculating *A*_*total*_(*t*) could be developed in future experiments to further refine the proposed dynamic method. For example, higher resolution sCT could be used to measure real values of *A*_*total*_(*t*). Nevertheless, using this dynamic method of surface area calculation, we demonstrate that *R*_*free*_ = *R*_*crystal*_ until effects inherent to growth within a pore structure become relevant to the growth kinetics, thus invalidating equation (3) (Stages 3–4). These effects are a consequence of microstructural changes in the pore network during precipitation (Supplementary Fig. S3 and Supplementary Table S1).

### Flow velocities

To explain the decrease of the growth rate during Stage 3, i.e. the divergent behavior between *R*_*free*_ and *R*_*crystal*_, the flow velocities, permeability and Peclet number (*Pe,* the ratio between diffusive and advective time scales, see Supplementary Information) were calculated at five times between the beginning and the end of the experiment (Table 1 and see Supplementary Information for details on the flow simulations). The decrease in permeability, during pore occlusion with barite, is more significant during Stage 3. This is unexpected since during Stage 2 crystals grow faster than during Stage 3, which suggests that during Stage 3 a more significant occlusion of flow paths occurs, and consequently, the growth rates decrease. The decrease of *Pe* indicates a shift towards transport-controlled growth kinetics. Nevertheless, the final *Pe* is 6000, three orders of magnitude higher than the value at which growth rates should be considered limited by transport (*Pe* ~ 1)^38^. Therefore, ion transport in the largest pores, which remain open and interconnected throughout the experiment (Videos 2 and 3), is expected to be sufficient to maintain the overall solution composition constant over the length of the column (*Pe* > 100)^38^.

**Figure 2:**
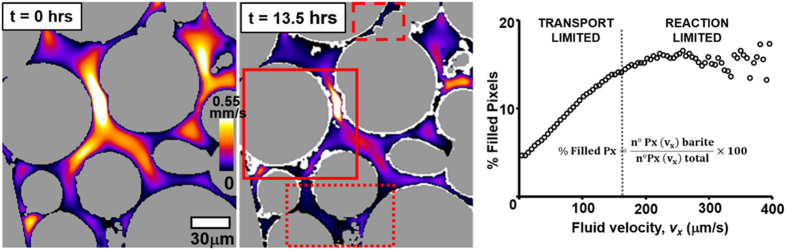
Distribution of flow velocities in a vertical cross section at the center of the column before growth and after 13.5 hours. SiO_2_ particles are represented in grey and barite in white. Color scale refers to flow velocities. Lines enclose areas where flow: remains fast during the entire experiment, whereby crystal density is high (continuous line); is fast at the beginning and slow after 13.5 hours, in contrast crystal density is low (dashed line); is slow at the beginning and after 13.5 hours, few crystals are observed (dotted line). The graphic shows the 3D statistical spatial relation between the flow velocities and barite precipitation.

The flow simulations revealed that at the pore scale flow velocities can diverge significantly, which is linked to the local and overall growth rates (Fig. 2). A larger density of crystals is found in pore-throats where the flow velocities remain fast throughout the duration of the experiment (continuous box). Lower crystal densities are found in regions where the flow velocities are initially fast but decrease with time (dashed box). These regions contrast with areas where flow is slow throughout the experiment (dot box) and fewer crystals are observed. This could be because in areas with slow flow the nucleation density is lower than expected or the growth rate is too slow to form crystals large enough to be detected within the resolution of this experiment. These regions represent <2% of the overall pore surface area. Consequently, since our dynamic surface area model assumes homogeneous nucleation density and growth rate, the estimated surface area is slightly overestimated, which ultimately results in a small overestimation of the growth rate. This error can contribute to the oscillation of calculated growth rates during Stage 2, however, within the experimental variability the error is not sufficiently large to invalidate equation 3.

A 3D statistical spatial correlation between the distribution of crystals and the distribution of flow velocities (Fig. 2) shows that at the end of the experiment more crystals are present in volumes where the flow was initially faster (see segmentation details in Supplementary Information). The increase of the percentage of pixels filled with barite as a function of the initial fluid velocity in those pixels is linear up to approximately 170 μm/s. Faster flow velocities do not seem to cause a significant change in the growth rate. Note that above 300 μm/s the points are scattered because there is less statistical data due to fewer pixels in each interval of flow velocities (Supplementary Fig. S2).

Our results are concordant with previous literature showing that mineral reaction rates in porous media are strongly dependent on permeability and local flow velocities^11^,^18^. A linear relation between dissolution rates and flow velocities, when the kinetics is transport-limited, has also been proposed based on reactive transport models^39^. Similarly, here we hypothesize that in subvolumes where the flow velocities are slower than approximately 170 μm/s growth is limited by transport, and above that velocity growth is limited by the reaction kinetics. Slower velocities may be insufficient to replenish the mineral-fluid interface with the same concentration of Ba^2+^/SO_4_^2−^ ions as the overall solution. Since during the experiment the flow velocity in some pore-throats decreases below 170 μm/s (Videos 2 and 3) we conclude that the overall decrease of growth rates during Stage 3 is caused by transport limitations in subvolumes of the column. This result is highly relevant as it is in contradiction with a growth rate limited by the reaction over the entire column, based on a high *Pe* during the experiment. Therefore, we conclude that parameters that represent the transport conditions averaged throughout a pore structure may not be sufficient to interpret mineral reaction rates in heterogeneous porous media that is highly affected by local properties.

To generalize, mineral growth rates in a permeable heterogeneous microporous structure is similar to that expected on free surfaces if a) growth is not limited by transport, not only throughout the structure but also in the subvolumes with reduced permeability; and b) the rate is normalized to the evolving surface area of the growing crystals, e.g. using the dynamic method presented here, equations (1, 2, 3). The two points are closely related since the method used to calculate *A*_*crystal*_(*t*) is only valid if the solution is homogeneous over the entire porous structure, which is not the case if growth is transport limited in subvolumes of the structure. The presented dynamic surface area model is sufficient to establish the links between the evolving microporous network, flow properties and different kinetic stages. Now that we stablished the importance of accounting for the dynamic reactive surface area of crystals, it is clear that future research should focus on better ways to measure or calculate the reactive surface area as a function of time. Furthermore, we suggest the development of directional correlation functions to analyze the link between flow velocities and mineral growth rates^40^,^41^. Such combination of numerical and experimental methods is crucial to validate predictive reactive transport models and calculate the timeframe of key geological processes.

## Methods

### Experimental setup

The experiment consisted of flowing a solution supersaturated relative to barite through a microporous quartz column. The column with inner and outer diameters of 1.6 mm and 3.0 mm, respectively, was prepared by filling 10 mm of the column length with a mixture of 95 weight % borosilicate glass beads (3–150 μm) and 5 weight % natural quartz particles (180–300 μm). Column and filling were sintered at 645 **°**C for 5 minutes, forming a solid porous structure. Using a tomographic scan of the dry column, it was determined that the different sizes of beads and quartz particles formed an irregular pore structure with pore diameters up to 122 μm (Supplementary Fig. S6) and 39% fully interconnected porosity.

Synchrotron X-ray radiation at beam line 13-ID-B of the Advanced Photon Source was used to generate one tomogram of the column every 24 minutes. A total of 3200 projections were acquired per scan over 360 degrees with a beam intensity of 22 keV. The volume imaged was 2.38 × 2.38 × 1.49 mm (8.4 mm^3^) with a voxel size 1.24 μm, of which 3 mm^3^ corresponds to the inside of the column. This volume contains 71.3 mm^2^ of SiO_2_ surface area exposed to the fluid. Two solutions, a 0.23 mM Na_2_SO_4_ and a 0.23 mM BaCl_2_, where continuously flowed into a 5 mL chamber with magnetic stirring, forming a solution with a saturation index (*SI*) of 2.1, as defined in equation (S2), where *a*_*Ba*_ and *a*_*SO4*_ are the activity coefficients of barium and sulfate, respectively, and *K*_*SP*_ is the solubility product of barite defined in PHREEQC-2. The experiment was performed at 21 ± 1 **°**C. The resulting solution was continuously fed to the column over the course of 13.5 hours with an initial flow rate of 60 mL/hr and a continuous pressure throughout the experiment applied by a Masterflex peristaltic pump from Cole-Parmer.

### Data analysis

Data was reconstructed using *IDL* software available at the beam line that generates stacks of 16-bit images, which were segmented using *Avizo 8* and *Fiji* (see segmentation detail in Supplementary Information). Reconstructed 3D stacks were segmented into three phases, each with a specific density that can be independently studied by thresholding their specific intensity: 1) *fluid*, fulfills the pore space; 2) *SiO*_*2*_, composes the solid matrix including glass beads, quartz particles and walls of the column; and 3) *barite crystals,* which nucleate on the SiO_2_ and grow into the fluid with time. The strong attenuation contrast between barite and SiO_2_ allows the quantification of the volume of barite precipitated at each time, which is used to calculate the average growth rate between scans.

### Simulation of flow velocities

The flow velocity field was simulated directly on voxelized images of the pore space resulting from segmentation of sCT scans^42^,^43^. In total we numerically analyzed five subvolumes obtained at different times: initial SiO_2_ porous structure at *t* = 0 hours and four subsequent stages with precipitated barite at *t* = 5.5, 8.5, 11 and 13.5 hours. Registered cubic subvolumes (edge length of 1.12 mm, corresponding to 900^3^ voxels) taken from the center of the column were used for all simulations. Within the initial subvolume the porosity was 35%, the maximum pore diameter was 89 μm and the average connectivity between pores was 7.3 pore-throats (see full statistical data for all subvolumes in Supplementary Information). Assuming creeping flow conditions (i.e., low Reynolds number (*Re*)), we numerically solve the so-called Stokes problem:


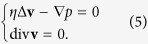


where ***v*** is the velocity vector, *η* is the viscosity of the fluid, and *p* is the pressure field. No-slip boundary conditions are applied to all fluid-pore wall interfaces. Prescribed pressure boundary conditions assuming a linear pressure drop of 1 Pa per voxel and a viscosity of 10^−3^ Pa/s were used to solve equation (5). The flow rate measured at the beginning of the experiment was 60 ml/h. It was assumed that the constant pulses generated by the peristaltic pump, i.e. constant rotation speed, kept the pressure at the inlet approximately constant during the length of the experiment as a progressive decrease of the flow rate was observed. The four faces of the cube parallel to the flow direction were treated as solid walls (no-flow boundaries). This approach causes a distortion of the flow velocities in the voxels immediately adjacent to the lateral surfaces of the cube relative to the experimental flow conditions. This effect is short-ranged and since we use a cubic domain’s size of 729 million voxels, which represents 62% of the total cross-sectional flow area, it is assumed to not affect the representativity of the overall experimental volume. Consequently, an adequate balance between representation of the experimental conditions and computational resources needed to solve the Stokes problem is achieved (see details in Supplementary Information).

## Additional Information

**How to cite this article**: Godinho, J. R. A. *et al*. The dynamic nature of crystal growth in pores. *Sci. Rep.* **6**, 33086; doi: 10.1038/srep33086 (2016).

## Supplementary Material

Supplementary Information

Supplementary Video 1

Supplementary Video 2

Supplementary Video 3

## Figures and Tables

**Table 1:** Properties of the pore structure after 0, 5.5, 8.5, 11 and 13.5 hours.

Time (hours)	0	5.5	8.5	11	13.5
Peclet number (×10^3^)	22.8	18.4	15.6	12.9	6.0
Permeability (×10^−4^)	5.49	4.50	3.88	2.33	1.47
Maximum velocity (μm/s)	712.2	700.9	695.6	653.0	611.1

Data corresponds to the 900 pixel side cube where flow velocities were simulated. Other statistics of the pore structure can be found in Supplementary Table S1.
