# Supplementary material for: The dynamic nature of crystal growth in pores
Source: Sci Rep. 2016 Sep 12;6:33086. doi: 10.1038/srep33086 (PMC5018885; doi:10.1038/srep33086)
Supplement: Supplementary Information [file srep33086-s1.pdf]

The dynamic nature of crystal growth in pores

Jose R. A. Godinho[*,1,2,3]*, Kirill M. Gerke*[4,5,6]*, Andrew G. Stack*[3]*, Peter D. Lee*[1,2]

[*] jose.godinho@manchester.ac.uk, +44 7487680526.

[1] School of Materials, The University of Manchester, M13 9PL, Manchester, UK.

[2] Research Complex at Harwell, Rutherford Appleton Laboratory, OX11 0FA, Harwell, UK.

[3] Chemical Sciences Division, Oak Ridge National Laboratory, PO Box 2008, MS-6110, Oak Ridge, TN 37831 USA.

[4] The University of Melbourne, Department of Infrastructure Engineering, Parkville, VIC, 3010, Australia.

[5] CSIRO Land and Water, Glen Osmond, PB2, SA 5064, Australia.

[6] Institute of Physics of the Earth of Russian Academy of Sciences, Bolshaya Gruzinskaya 10, Moscow, 107031, Russia.

The supporting information contains 6 figures, 1 table, 4 equations and 11 pages.

Video 1: Sequence of 2D images of a cross section of the column at 7 growth times (0, 4, 6, 8, 10, 12 and 13.5 hours. Barite is the brightest fraction, SiO_2 particles are light grey (irregular particles are quartz and circular particles are glass beads), pores are dark grey, and the air trapped inside glass beads is the darkest fraction. The flow direction is from the top to the bottom of images. Each 2D image was filtered using non-local means filter.

Video 2: 3D visualization of the inside of the column 1) before growth and after 13.5 hours, pore space is transparent, SiO_2 particles are grey and barite crystals are red; 2) before growth and after 13.5 hours, pore space is colored based on the fluid velocity. Note the homogeneous distribution of barite throughout the structure, and a shift to darker colors in the rendering of the flow velocity between 0 and 13.5 hours that means a decrease of the local flow velocities.

Video 3: Flow velocities at the center of the column. Pore-throats with flow velocities slower than 85 μm/s are not represented. Note that although the average flow velocity decreases substantially and several regions have slow flow, the velocities in the main flow paths remain fast.

Segmentation

Segmentation of the **initial SiO_2 porous structure** from a 16-bit stack of images corresponding to the dry column before the experiment: 1) “Anisotropic diffusion 3D filter” (Threshold = 3000, Iterations = 10); 2) Threshold the fraction corresponding to the SiO_2; 3) Mask the space outside the edge of the column. 4) “Fill holes 3D” function to remove air bubbles inside the beads.

Segmentation of the **total volume of barite** for each scan from 16-bit stacks of images: 1) Align the stack with the initial data set (fine registration function); 2) Mask the data with the initial SiO_2 structure; 3) Threshold the brightest fraction that corresponds to barite. Particles of only one pixel were considered noise, thus not accounted as barite. This segmentation method can cause an overestimation of the volume if pixels that are accounted as barite are only partially filled with crystals, or an underestimation of the volume if the intensity of pixels that contain a small amount of crystals are below the applied threshold.

Calculation of **$A_{pore}(t)$ for each scan**: 1) five pixel dilation of the solid phase composed by silica and barite, 2) five pixel erosion of the result from 1, 3) overlap the result from 2 with a thresholded image of just barite crystals in each scan. The number of pixels of the overlapped image is multiplied by the surface area of the face of a voxel (i.e. a square), 1.54 μm^2. This sequence of operations identifies the pore-throats that are filled with crystals (blue in Supplementary Figure S1). The resulting area corresponds statistically to the surface area of pores that are occluded, thus no longer contribute to the growth rate. Finally, this surface area is subtracted from the initial A_{pore}. Note that due to the lack of resolution it is not possible to account for the decrease of the reactive surface area caused by the lateral overlapping of two crystals. It is important to highlight that this is a statistical method aimed to minimize the error associated with overgrowth along restricted directions when applying equation (3) (see Supplementary Figure S1).

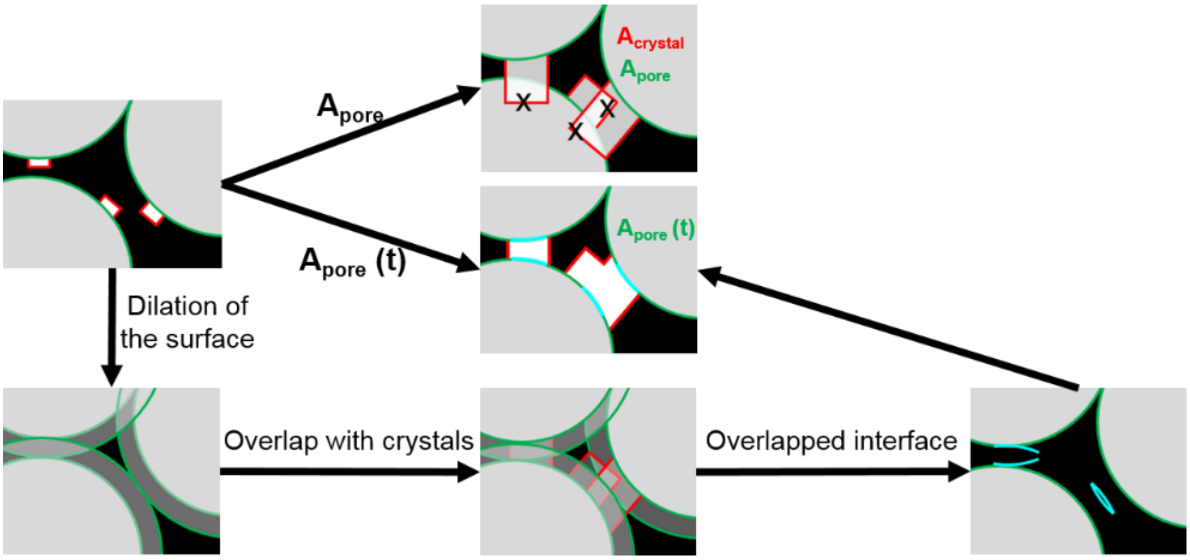

Supplementary Figure S1: 2-d schematic representation of how $A_{crystal}(t)$ was calculated for each stage.

Graphic in Figure 2: A distance map from the surface of the pore structure was generated using a 3-4-5 chamfer transform in Avizo 8, using the initial pore structure. A threshold was applied to the color map in order to select the layer closest to the surface (LCS) of the beads that is 12.4 μm thick. The resulting binary stack was multiplied by the stack of the flow field at t = 0 hours. The results consist of the flow velocities in the LCS, further called dataset A. The stack's histogram was divided in 100 groups by color intensity, each corresponding to an interval of fluid velocities (v_x). The total number of pixels in each group corresponds to $Px\ (v_x)\ total$ in equation (S1). The color scale was normalized using the maximum flow velocity corresponding to the original flow field (Table 1). Additionally, dataset A was multiplied by a stack containing only barite corresponding to the end of the experiment. The result is a stack of barite crystals for which each pixel is colored based on the fluid velocity in the original dataset A. Each interval (v_x) contains the number of crystals that grow in a specific range of flow velocities ($Px\ (v_x)\ barite$). Circular points in the graphic of Figure 2 correspond to the percentage of pixels filled with in each interval of flow velocities, (S1).

$$\%\ \text{Filled Px} = \frac{\text{Px}\ (\text{v}_\text{x})\ \text{barite}}{\text{Px}\ (\text{v}_\text{x})\ \text{total}} \times 100 \quad \text{(S1)}$$

The distance LCS=12.4 μm was chosen because it encompasses the region where crystals grow, i.e., 12.4 μm is about the size of the largest crystals. This reduces the

influence of regions within the pore space where crystals cannot reach within the timeframe of the experiment, e.g., the center of large pores where flow velocities are typically faster than in pore-throats. The variable *% Filled Px* presented here was chosen because from our data the amount of barite grown at each scan cannot be directly related to the overall flow velocity since the distribution of flow velocities is not proportional throughout the porous structure (Supplementary Figure S2). Consequently, results in Figure 2 are necessarily normalized to the total number of pixels corresponding to the same velocity group within the LCS. Nevertheless, it is hypothesized that *% Filled Px* is proportional to the amount grown in a region of the pore space, which allows linking statistically the overall growth rate to the velocity field. Therefore, in the discussion of Figure 2 it is assumed that higher values of *% Filled Px* correspond to faster growth rates.

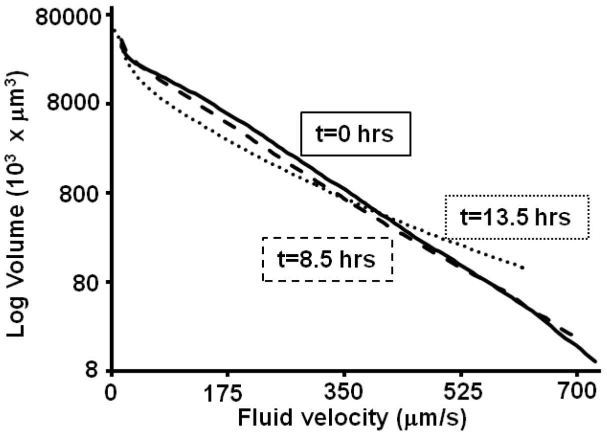

Supplementary Figure S2: Volume distribution of pixels with specific flow velocities at *t* = 0 hours (continuous line), *t* = 8.5 hours (dashed line) and *t* = 13.5 hours (dotted line). Data corresponds to the 900 pixel side cube where flow velocities where calculated.

Pore size distribution

To characterize the changes in the porous structure we use a conventional analysis based on pore and pore-throat size distributions.[43] The method of maximal inscribed spheres,[44] was used to extract pore and pore-throat sizes. The method consists of the following 1) for each voxel representing the pore space, spheres are inscribed with a radius from the centre of the voxel to the nearest pore wall; 2) all redundant spheres included into larges spheres are deleted; 3) the resulting spheres are clustered according to their sizes so that local maxima define pores and the rest of the spheres

are assigned as their descendants; 4) the largest sphere among those sharing the same ancestors is defined as a pore-throat. In all steps we closely follow Dong and Blunt,[43] except that we do not randomize inscribed sphere sizes and always chose the upper limit radius (R_{right}) for all spheres. The latter ensures that the comparison between different subvolumes is consistent and unaffected by randomization. Inlet and outlet throats are removed from the analysis. The average pore connection number was computed for each subvolume and represents the average number of pore-throats connected to pores.

The number of individual pores and pore-throats increase with time (Supplementary Table S1), mostly due to an increase of the number of pores smaller than 30 μm diameter and pore-throats smaller than 15 μm diameter (Supplementary Figure S3). Above those diameters the number of pores and pore-throats decrease with time. The creation of small pores is likely to be related to the entrapment of small volumes by crystals growing on adjacent beads.

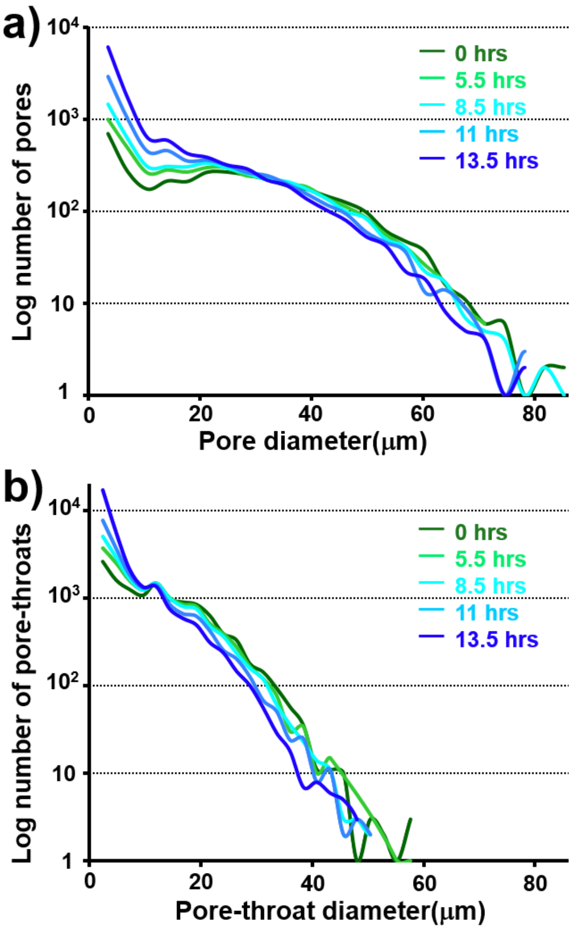

Supplementary Figure S3: Number of pores **(a)** and pore-throats **(b)** that have a specific diameter at 5 different times.

<u>Spatial distribution of barite after 13.5 hours</u>

The dispersion of barite throughout the column was quantified using the function "Z Project – sum slices" on binary stacks containing just the barite phase, perpendicular to the flow direction (Supplementary Figure S2 -a) and parallel to the flow direction (Supplementary Figures S2 -*a* and -*b*). Z project sums the amount of barite pixels for each plane (in this case XY or XZ for each pixel position along all the planes of the stack. Similar to a radiograph, the result is a 2D image where brighter colors correspond to larger amounts of barite in that specific position along a line perpendicular to the specific plane analyzed (Supplementary Figures S4 -*a* and -*b*). Quantification of the amount of barite was achieved by normalizing the color map to the amount of pixels with barite along each profile (continuous line – X axis, dotted line – Y axis, dashed line – Z axis). Over the length of the column in all directions the amount of barite oscillate within 13% of the average value (131), although these oscillations are localized (e.g. region marked with a circle). These local variations are possibly associated to regions with larger pores. Note that in Supplementary Figure S4-b the central region is brighter than the left and right sides, which is a consequence of the column shape, i.e. a perpendicular line at the center of the column crosses more porous structure than a line at the edge of the sample, thus more barite should be found. Therefore, the analysis of this Figure is limited to comparing points along vertical lines where the density of the porous structure is constant, e.g. dashed line.

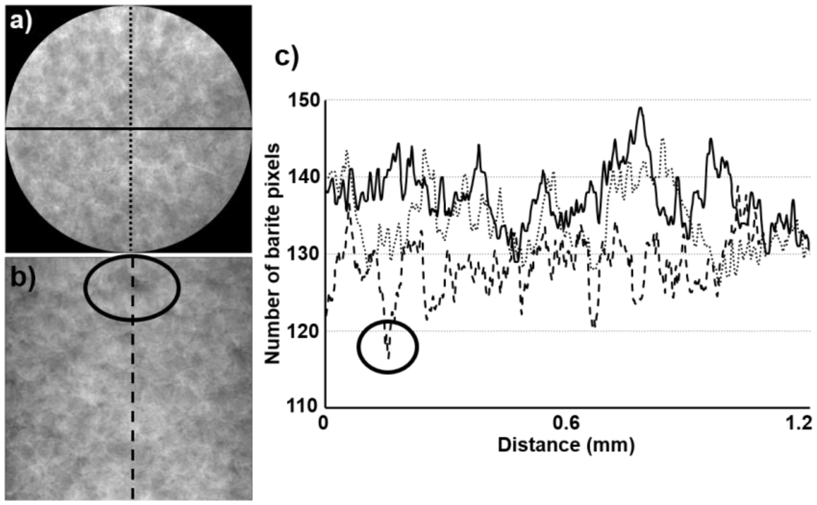

Supplementary Figure S4: Quantification of barite dispersion throughout the column after 13.5 hours. **a)** Top view of the column; **b)** side view of the column; **c)** quantification of the number of pixels of barite along the center lines describes in *a* and *b*. Brighter colors in *a* and *b* correspond to higher amounts of barite. Circles in *b* and *c* mark a region with less barite.

<u>Comparison of the volume of barite calculated using different methods</u>

At 8.5 hours the volume of barite measured by sCT is slightly lower than that expected on free surfaces calculated using *A(t)* and R_{free} in equation (2) and using the total surface area of our sample. A direct comparison between V_{free} and $V_{crystal}$ can be established because both variables are calculated as a function of time. The hypothesis that during Stages 1-2 the growth kinetics is not significantly affected by pore restrictions is strength by the observation that V_{free} and $V_{crystal}$ are similar between 4 and 8.5 hours. For the purpose of upscaling the results, e.g., to estimate the volume of barite that would precipitate in a geological formation of known approximate surface area, it is relevant to back calculate the volume of crystals estimated to precipitate in our porous structure (V_{pore}) using $R_{crystal}$. V_{pore} follows a similar trend to V_{free} during the initial 8.5 hours (inset of Supplementary Figure S5), and both $V_{crystal}$ and V_{pore} significantly diverge from V_{free} after 8.5 hours. A very relevant observation is that even if the overall R_{pore} is higher than R_{free}, at 13.5 hours the volume precipitated is significantly lower than the amount expected on a free surface.

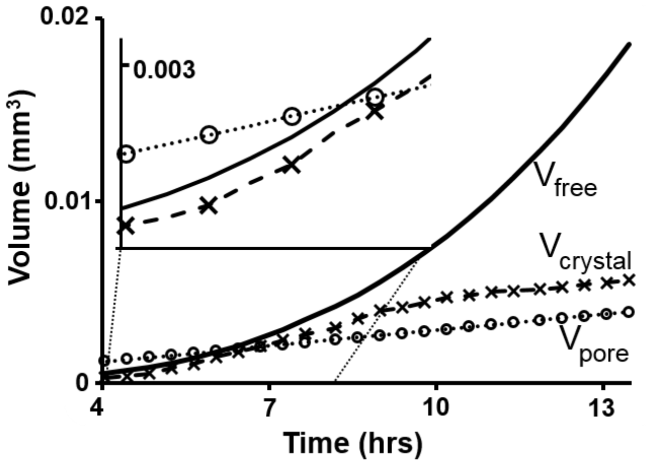

Supplementary Figure S5: Variation of the total volume of barite as a function of time. Dashed line (**X**) corresponds to values measured in this experiment directly from sCT. Continuous line correspond to the volume expected on free surfaces for the same solution composition using a nucleation density *N*=0.29 crystals/μm^2, the initial pore surface area

A_{pore}=71.3 mm^2, and an empirical equation to calculate the volume of single crystals *V(t)*.[33] Dotted lines (**o**) correspond to volumes back calculated from R_{pore} and using A_{pore}. Stage 2 when R_{free} and $R_{crystal}$ are similar is zoomed in as an inset. Data corresponds to the total pore volume within a field of view, 3 mm^3.

Methods

The mixing chamber was connected to the column by 15 cm of tygon tube 3 mm diameter. This is the minimum length technically possible that minimize precipitation before the solution enters the column, whilst allowing 360 degrees rotation of the sample.

$BaCl_2$ and Na_2SO_4 solutions were prepared by dilution of 0.01 M stock solutions. Stock solutions were prepared from p.a. anhydrous Na_2SO_4 (Acros) and laboratory grade anhydrous $BaCl_2$ (Fischer Scientific).

$$SI = log \frac{a_{Ba} a_{SO_4}}{K_{sp}} \qquad (S2)$$

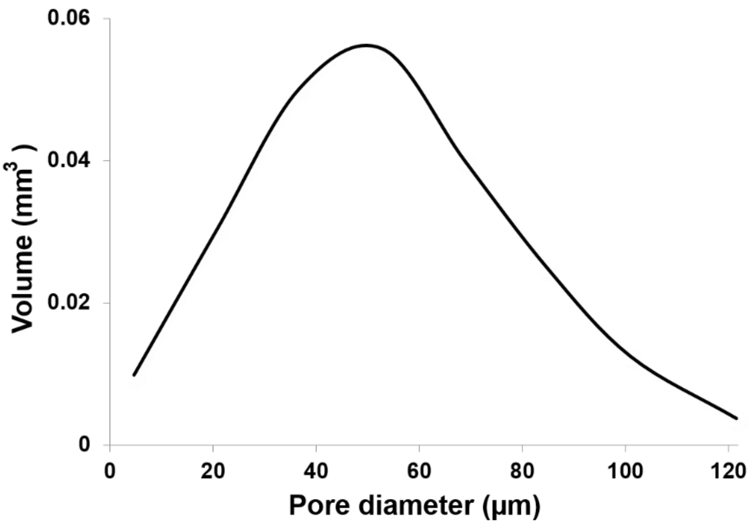

Supplementary Figure S6: Volume corresponding to each pore diameter at the beginning of the experiment measured by the method of inscribed spheres (function “thickness” in boneJ)[45].

Numerical simulation of flow velocities

The Stokes problem was iteratively solved using the finite-difference scheme with pressure discretized at the center of the voxels and velocities at the boundaries of the voxels. This is known to produce accurate results while having low demand for computational resources.[46] An in-house C++ parallelized Stokes solver based on artificial compressibility method was used for all computations.[47] The code was tested on sphere packings and produce an accuracy comparable with D3Q19 TRT lattice-Botzmann simulations.[48] Model solutions only deviated significantly from analytical solutions for low resolved sphere packings where flow was controlled by connections between voxel edges,[49] which was not the case for all stages of our experiment (see pore-throat size distributions in Figure S5). All simulations were iterated until criteria based on imbalance for both continuity[43] ($<10^{-6}$) and motion equations have reached a plateau (less than 0.001% change in last 20 iterations). This required from 13 x 10^3 to 12 x 10^4 iterations depending on the subvolume, and ensured that the computer-specific limit, beyond which the difference between two consecutive iterations is small and random, was reached. After convergence of the velocity and pressure fields, the permeability was calculated using the Darcy's Law.[38]

To study the correlation between fluid flow velocity and barite precipitation, permeability values are insufficient and the full velocity field is required. To calculate velocity fields representative of those in the sample at different times, the pressure boundary conditions were calculated using the permeability and initial flow rate to solve Darcy's law. First, equation (5) was solved for all five subvolumes to obtain permeability and velocity fields.[50,51] The permeability is a property of the pore geometry and the formulation of the Stokes problem depends only on the voxel resolution. The latter means that correct permeability values are obtained for all subvolumes with these pressure boundary conditions, which are different from experimental ones. Second, using the measured flow rate of 60 ml/h the unknown pressure drop conditions in the initial subvolume (t = 0 hours) were calculated. The continuity condition was applied, thus it was assumed that the flow rate is constant throughout the column. Finally, the velocity fields in the five subvolumes were rescaled to correct pressure drop in the initial subvolume.

Calculation of the Reynolds and Peclet numbers

To characterize transport conditions throughout the porous network, the Peclet number (*Pe*) and Reynolds number (*Re*) where calculated using equations (S3) and (S4). *Pe* is the ratio between diffusive and advective time scales, where *D* is the molecular diffusion coefficient of barium in solution, U_v is the Darcian velocity, θ the total porosity measured experimentally by sCT and *L* is the characteristic length. For packings of mono and polydisperse spheres (our sample is close to such packings) it is common to use so-called Sauter diameter,[52] calculated from $6 \sum Vp / \sum Ap$, where *Vp* is the volume of each particle and *Ap* is the particle surface area. In our calculation we did not divide the solid structure into separate particles (e.g. using a watershed algorithm), but rather used the total volume and total surface area of the solids measured from sCT. Calculated this way, the characteristic length is similar to that calculated in previous work for non-granular solid materials.[44,46] *Re* was calculated to ensure that creeping flow conditions are applied to our experimental and modeling setup, where ν is the kinematic viscosity. *Re* values during the experiment (Supplementary Table 1) are low enough to ensure that Stokes flow conditions are satisfied and calculations using equation (5) are valid.

$$Pe = \frac{U_v.L}{D.\theta} \quad \text{(S3)}$$

$$Re = \frac{U_v.L}{\nu} \quad \text{(S4)}$$

Supplementary Table S1: Properties of the porous structure after 0, 5.5, 8.5, 11 and 13.5 hours, as complement of Table 1. Data corresponds to the 900 pixel side cube where flow velocities were simulated.

**Time**	**0**	**5.5**	**8.5**	**11**	**13.5**
Reynolds number (x10^{-4})	21.4	17.3	14.6	9.8	5.7
Number of pores	3476	4245	4980	7190	11294
Number of pore-throats	12553	14857	16578	19440	30343
Average pore connection number	7.3	7.1	6.8	5.5	5.4

References

44. Menke, H. P., Bijeljic, B. R., Andrew, M. G. & Blunt, M. J. Dynamic three-dimensional pore-scale imaging of reaction in a carbonate at reservoir conditions. *Environ. Sci. Technol.* **49**, 4407-4414; DOI:10.1021/es505789f (2015).
45. Dougherty, R. & Kunzelmann, K. Computing local thickness of 3D structures with ImageJ. *Microsc. Microanal.* **13**, 1678-1679; DOI:10.1017/S1431927607074430 (2007).
46. Mostaghimi, P., Bijeljic, B. & Blunt, M. J. Simulation of flow and dispersion on pore-space images. *SPE J* **17**, 1131-1141; DOI:10.2118/135261-pa (2012).
47. Chorin, A. J. A Numerical Method for Solving Incompressible Viscous Flow Problems. *J. Comp. Phys.* **135**, 118-125; DOI:10.1006/jcph.1997.5716 (1997).
48. Khirevich, S., Ginzburg, I. & Tallarek, U. Coarse- and fine-grid numerical behavior of MRT/TRT lattice-Boltzmann schemes in regular and random sphere packings. *J. Comp. Phys.* **281**, 708 -742; DOI:10.1016/j.jcp.2014.10.038 (2015).
49. Manwart, C., Aaltosalmi, U., Koponen, A., Hilfer, R. & Timonen, J. Lattice-Boltzmann and finite-difference simulations for the permeability for three-dimensional porous media. *Phys. Rev. E* **66**, 016702; DOI:10.1103/PhysRevE.66.016702 (2002).
50. Adler, P. M., Jacquin, C. G. & Quiblier, J. A. Flow in simulated porous media. *Int. J. Multiphase Flow* **16**, 691; DOI:10.1016/0301-9322(90)90025-E (1990).
51. Martys, N. S., Torquato, S. & Bentz, D. P. Universal scaling of fluid permeability for sphere packings *Phys. Rev. E* **50**, 403; DOI:10.1103/PhysRevE.50.403 (1994).
52. Daneyko, A., Höltzel, A., Khirevich, S. & Tallarek, U. Influence of the particle size distribution on hydraulic permeability and eddy dispersion in bulk packings. *Anal. Chem.* **83**, 3903-3910; **DOI:**10.1021/ac200424p (2011).
