# Supplementary figures and images for: The dynamic nature of crystal growth in pores
Source: Sci Rep. 2016 Sep 12;6:33086. doi: 10.1038/srep33086 (PMC5018885; doi:10.1038/srep33086)

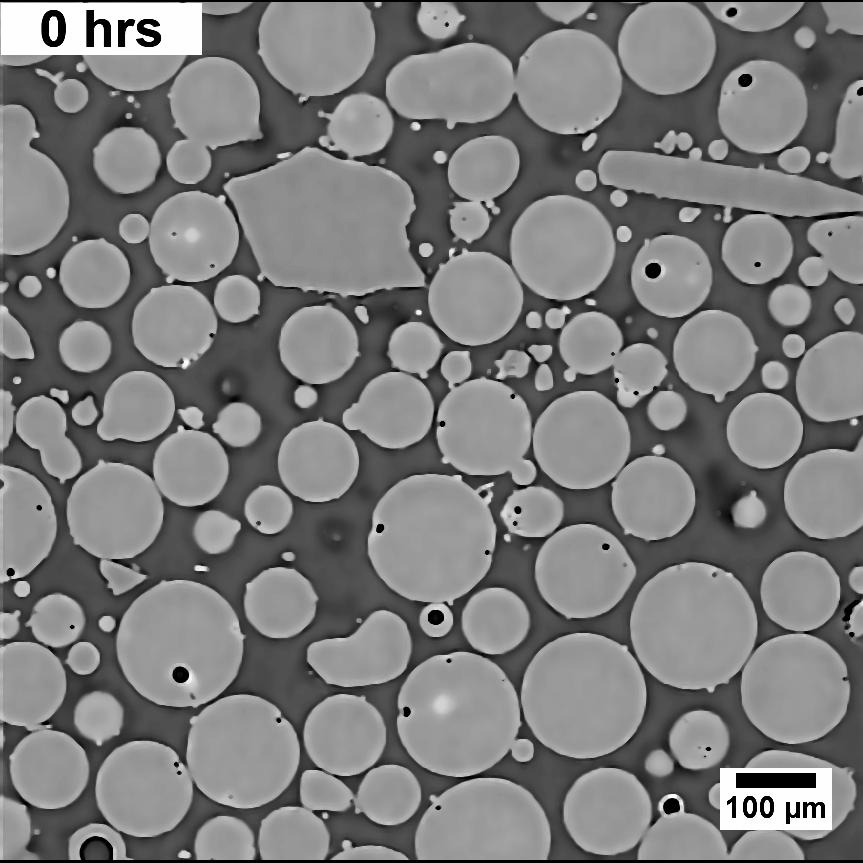

Supplement: Supplementary Video 1 [file srep33086-s2.gif]
